# Restoration of brain dystrophin using tricyclo-DNA ASOs restores neurobehavioral deficits in DMD mice

**DOI:** 10.1016/j.omtn.2023.04.007

**Published:** 2023-05-25

**Authors:** Muthukumar Karuppasamy, Matthew S. Alexander

**Affiliations:** 1 Department of Pediatrics, Division of Neurology at the University of Alabama at Birmingham and Children’s of Alabama, Birmingham, AL 35294, USA; 2 UAB Center for Exercise Medicine at the University of Alabama at Birmingham, Birmingham, AL 35294, USA; 3 Department of Genetics at the University of Alabama at Birmingham, Birmingham, AL 35294, USA; 4 UAB Civitan International Research Center (CIRC), at the University of Alabama at Birmingham, Birmingham, AL 35233, USA; 5 UAB Center for Neurodegeneration and Experimental Therapeutics (CNET), Birmingham, AL 35294, USA

Duchenne muscular dystrophy (DMD) is a progressive, X-linked neuromuscular disorder that is caused by pathogenic variants in the dystrophin gene resulting in the loss of the production of a functional dystrophin protein. DMD patients suffer from muscle degeneration, respiratory weakness, loss of ambulation by their teenage years, and cardiomyopathy ultimately leading to patient death by their third decade. Steroid regimens have extended DMD patient lifespan and delayed DMD patient symptoms, but no cure currently exists. Dystrophin replacement gene therapies (both micro- and mini-dystrophin) along with exon-skipping antisense oligo (ASO) compounds have shown promise in delaying disease progression.^1^^,^^2^^,^^3^^,^^4^ DMD exon-skipping compounds function to “skip” over or bypass the mutant transcript and restore the DMD reading frame resulting in the production of a novel Becker muscular dystrophy (BMD)-like transcript and dystrophin protein product. Several exon-skipping drugs have been approved by the US Food and Drug Administration for DMD; however, these compounds usually have low corrected dystrophin protein muscle expression.^5^ Newer exon-skipping compounds and delivery strategies are currently being developed to improve both dystrophin translation amount and systemic biodistribution of the exon-skipping compounds.

The large dystrophin (human gene symbol *DMD*) isoform (Dp427) is expressed in skeletal and cardiac muscle; however, expression of the large Dp427 isoform and multiple smaller dystrophin protein isoforms exist in the brain, some of which are transcribed via a brain-specific promoter. Assessments of the expression of human dystrophin isoforms have revealed key timing and isoform requirements for the role of brain dystrophin isoforms in impacting cognitive function.^6^ Questions still remain on the exact nature and impact of the large Dp427, and the functional requirements for the smaller Dp140 and Dp71 brain dystrophin isoforms that have all been implicated in dystrophin cognitive function. Previous work from this group demonstrated safety and therapeutic efficacy in the ability to restore dystrophin transcript and subsequent protein levels in the *mdx* (dystrophin exon 23 nonsense mutation) in multiple skeletal muscle groups and whole heart tissue using a 13-mer tricyclo-DNA AON construct following 12 weeks of dosing.^7^ These studies demonstrated the ability of these tcDNA constructs to cross the blood-brain barrier (BBB) penetration for low-level dystrophin correction; however, the extent of the functional improvement and consequences of this brain-mediated correction were not extensively evaluated.

**Figure 1:**
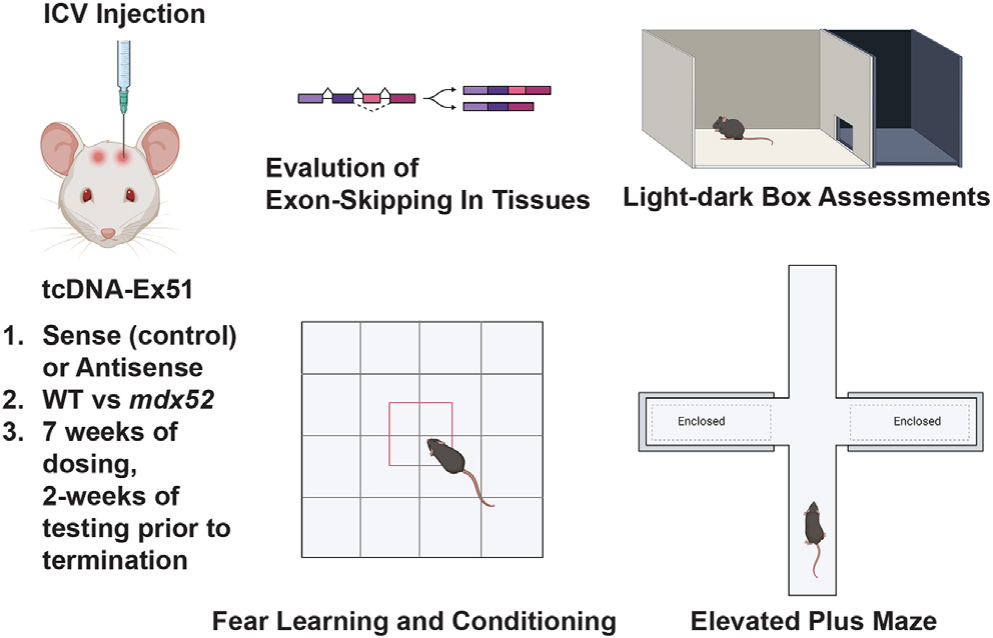
General representation of the Goyenvalle et al. study with key highlights Intracerebroventricular (ICV) administration of the tcDNA AON constructs in the 6- to 8-week-old wild-type or *mdx52* mice. Key evaluation of the exon-skipped mRNA transcript is essential for evaluation of the amount of tcDNA that corrects the dystrophin reading frame in the regions of the brain. Behavioral testing was performed 7 weeks post ICV injection. Fear learning and conditioning responses were recorded. Light-dark and elevated plus maze anxiety tests were also performed prior to mouse experiment termination.

In the current study, Goyenvalle et al. demonstrate that tricyclo-DNA ASOs are capable of the partial restoration of the large brain dystrophin isoform (Dp427) in *mdx52* mutant mice.^8^ Previous work has demonstrated that in addition to muscle defects, *mdx52* mice develop social anxiety, elevated fear responses, and impaired associative fear learning (Figure 1). The authors performed a single intracerebroventricular administration of tricyclo-DNA antisense oligonucleotides targeting exon 51 (tcDNA-Ex51) that restored between 5% and 15% of dystrophin protein in the hippocampus, cerebellum, and cortex of the brain 7–11 weeks post injection. The tcDNA-Ex51 treated mice showed improved anxiety phenotypes in the elevated plus maze responses but only partial improvements in emotional reactivity in the light/dark choice test. The overall fear response was also improved in the tcDNA-Ex51-treated *mdx52* mice compared with tcDNA-Ex51 sense oligo control cohorts. Close examination of the tcDNA-Ex51-treated *mdx52* mice showed partial improvement of auditory-cued fear conditioning in which the treated dystrophic mouse cohorts showed improvements in fear memory recall. Biodistribution profiling of the tcDNA-Ex51 revealed a homogeneous distribution of dystrophin exon-skipping, which was different from the authors’ previously reported evaluation of tricyclo-DNA antisense exon-skipping in the *mdx* (exon 23) mouse model, suggesting the unique biodistribution constraints of each individual ASO.^9^ Further modifications into systemic ASOs capable of BBB penetration leading toward the systemic restoration of dystrophin protein at substantial levels may lead to higher amounts of dystrophin restoration and improved dystrophic fear responses. Additional exploration into the longevity of the tcDNA-Ex51-restored dystrophin in aged mice and the use of tcDNA ASOs targeting other dystrophin exons in DMD mouse models and human DMD muscle cell lines could yield insight into the future application of the tcDNA technology toward DMD clinical trials.
